# Visualisation of Amphetamine Contamination in Fingerprints Using TOF-SIMS Technique

**DOI:** 10.3390/ma14216243

**Published:** 2021-10-20

**Authors:** Małgorzata I. Szynkowska-Jóźwik, Elżbieta Maćkiewicz, Jacek Rogowski, Magdalena Gajek, Aleksandra Pawlaczyk, Marcel de Puit, Andrzej Parczewski

**Affiliations:** 1 Faculty of Chemistry, Institute of General and Ecological Chemistry, Lodz University of Technology, Zeromskiego 116, 90-924 Lodz, Poland; elzbieta.mackiewicz@p.lodz.pl (E.M.); jacek.rogowski@p.lodz.pl (J.R.); magdalena.gajek@edu.p.lodz.pl (M.G.); aleksandra.pawlaczyk@p.lodz.pl (A.P.); 2 Digital Technology and Biometrics, Netherlands Forensic Institute, Laan van Ypenburg 6, 2497 GB Den Haag, The Netherlands; m.de.puit@nfi.nl; 3 Department of Chemical Engineering, Faculty of Applied Sciences, Delft University of Technology, Van der Maasweg 9, 2629 HZ Delft, The Netherlands; 4 Faculty of Chemistry, Jagiellonian University, Gronostajowa 2, 30-387 Cracow, Poland; parczews@chemia.uj.edu.pl

**Keywords:** fingerprint, TOF-SIMS, amphetamine visualisation, lifting tape, black powder

## Abstract

Time-of-flight secondary ion mass spectrometry (TOF-SIMS) was applied to detect traces of amphetamine on fingerprints. In the present study, three different lift tapes and latent powder fingerprints were tested. The obtained results show that it is possible to identify traces of a drug as well as its distribution over the tested fingerprint after its transfer from the primary base onto an adhesive lifter (secondary base). Moreover, images obtained by the TOF-SIMS technique enable the observation of very small areas of the analysed fingerprint as well as the identification of micro-objects (residues of a contaminant) that were left on the fingerprint. The use of the black latent fingerprint powder did not interfere with the TOF-SIMS analysis, which makes it possible to effectively use this technique to study the traces of substances on the revealed fingerprints.

## 1. Introduction

Papillary ridges are structures in the skin that are part of human fingers, palms, and feet. They are formed by the third to fourth month of embryogenesis and possess individual characteristics, which do not naturally change throughout one’s life. An impression left by the friction ridges of the human finger is known as a fingerprint, which may be transferred from the finger to other items in the form of a latent or patent print. The imprinted patterns can be related to the fingers, palms, and feet of an individual. As such, fingerprints can be used as a perfect tool for human identification or individualisation. Fingerprint identification or hand print identification, also known as dactyloscopy, is a very important field of forensic science, as it is proof of contact [1,2,3].

Fingerprints have distinct patterns that also hide lifestyle clues. Human skin is covered by natural excretion produced by glands. The chemical composition of fingermark residue differs from the general chemical composition of sweat because it contains a complex mixture of compounds coming from different glands (not only from the eccrine ones). Additionally, fingermark residues can also contain many contaminants such as cosmetics, food residue, dead cells, and skin oils, which contain residues of drugs or various chemicals and their metabolites present in the body [4,5]. Therefore, it is possible to determine whether a person is a smoker or takes drugs [6,7,8]. The presence of trace amounts of substances that do not exist in natural skin excretion may also be caused by the mere contact of the finger with some products (tobacco, drugs, etc.) or even by handshakes between a smoker and a non-smoker [8,9]. The last few years have shown an increasing application of various chemical imaging techniques in forensic science [10,11,12,13,14,15], especially in detecting exogenous materials present on fingermarks, such as particles of nicotine [9], gunshot residues [16,17,18,19], illicit drugs [6,7,8,18,19,20,21,22,23], caffeine [24,25], blood [26], explosive RDX residues [20,27], or in determining the sequencing of fingerprint and ink signals on documents [28,29].

Latent fingerprints are usually developed in situ at a crime scene by the application of powders. There are many types of latent fingerprint powders that are used by forensic technicians depending on e.g., the type of substrate and the quality of fingerprints [30]. After the treatment with powders, fingerprints have to be photographed and are often lifted with special adhesive tapes to transfer and preserve the details of the fingerprint. Nowadays, a wide range of different types of lifting materials, which are designed for lifting fingerprints from a range of surfaces, is commercially available.

In the previous study, we demonstrated time-of-flight secondary ion mass spectrometry (TOF-SIMS) as a potential tool in forensic research, especially in chemical investigations of fingerprints and the detection of traces of substances that do not exist in natural excretion, derived from crime scenes, for example metal-containing compounds, gunpowder residues, or arsenic [16,21,31]. The technique was also used in the visualisation and analysis of fingerprints contaminated with amphetamine drugs, which were imprinted on selected substrates (plates of steel, aluminium, brass, and glass). TOF-SIMS measurements carried out for that study were made directly on these substrates [20]. The size of the substrate is a limiting factor, as the vacuum chamber can only hold smaller items. However, at crime scenes, visualised fingerprints are often lifted with some sort of tape. Other authors described a comparative analysis of fingerprint residue with the use of a suite of relevant analytical techniques, proving that TOF-SIMS was very selective, showing reproducible differences between the donors of fingerprints by imaging a fingerprint in situ [32]. It was also demonstrated that SIMS imaging of fingerprints can provide a sufficient ridge and fingerprint minutiae detail to allow its use for forensic identification purposes [33]. Conventional SIMS has low sensitivity for high-mass molecules. For this reason, MeV SIMS, which is a technique complementary to SIMS, has recently re-emerged. It uses an MeV (rather than keV) primary ion beam [34,35,36]. MeV-SIMS has proved to be useful to determine the order of sequence of a doped fingerprint and an ink entry [28].

In the study presented here, TOF-SIMS was applied in the detection and visualisation of traces of amphetamine left on fingerprints. To the best of our knowledge, this work shows for the first time that secondary ions mass spectrometry can be applied as one of the steps of fingermarks studies and development in order to possibly enhance the visualisation and enable the detection of psychoactive substance even when the latent fingerprint powder has already been powdered. The scenario proposed by us includes the trace detection of amphetamine from fingerprints, fingerprints lifted by tape, and fingerprints powdered with fingerprint powder as well as from fingerprints lifted from fingerprint powder. The investigated steps seem to be a standard procedure typically used at the crime scene.

An amphetamine-contaminated fingerprint was deposited directly on a glass slide/surface. These fingerprints were treated with black latent fingerprint powder and lifted by tape in order to mimic the process usually followed at a crime scene.

## 2. Materials and Methods

### 2.1. Preparation of Samples

The sample of amphetamine used in the present investigation was obtained from the Faculty of Chemistry, Jagiellonian University, Cracow, Poland. Then, the substance was used to imitate the process of transfer of trace amounts of an exogenous contaminant from a finger onto a tape and its subsequent revealing by the latent fingerprint powder.

At first, an amphetamine reference sample was prepared. A tablet of amphetamine was formed in a tableting machine under pressure of about 7 tons. This tablet was analysed using TOF-SIMS in order to gather information about the type of secondary ions present for the studied reference material. Then, a volunteer left a fingerprint that had been contaminated with a small amount of amphetamine powder. The palms and fingers of the volunteer were washed and dried beforehand.

Then, the fingerprints contaminated with amphetamine were deposited on a glass and on three different adhesive materials employed for the transfer process of the fingerprints. The studied tape material consisted of commercially available Scotch adhesive tape, Remco fingerprint tape, and Filmolux fingerprint film. The last two materials are typically used by forensic technicians. TOF-SIMS spectra and ionic images of the tested adhesive materials (new, unused tapes and with a fingerprint contaminated with amphetamine) were created.

The criteria of choice of the adhesive material were based on the quality of the obtained fingerprints contaminated with amphetamine after the transfer of the fingerprints using the tested adhesive materials. Finally, the fingerprint contaminated with amphetamine was placed on the glass surface (analogous to the first stage), and then, the fingerprint was revealed with a black fingerprint powder (Stanimex, Lublin, Poland) and transferred using the previously chosen fingerprint tape. TOF-SIMS spectra and ionic images were made for both the latent fingerprint powder (in the form of a tablet, pressed in the same way as the studied amphetamine substance) and the fingerprint contaminated with amphetamine and revealed with the above-mentioned powder.

### 2.2. Instrumentation and Instrumental Parameters

The secondary ion mass spectra and images for the samples were recorded using a TOF-SIMS IV mass spectrometer manufactured by ION-TOF GmbH (Muenster, Germany). During the mass spectra measurement, the analysed area was irradiated with pulses of 25 keV Bi^3+^ ions at a 10 kHz repetition rate and an average ion current of 0.2 pA. The time of spectra acquisition was 30 s. The instrument was equipped with a Bi liquid metal ion gun and a time-of-flight mass analyser. Mass resolution was typically greater than 4000 at *m*/*z* 29 with the primary ion pulse width of 1 ns. Secondary ion mass spectra were recorded from an approximately 100 mm × 100 mm area of the analysed surface. TOF-SIMS images were recorded by scanning the surface of the sample with a pulsed beam of Bi^3+^ ions. The analysed area was 500 × 500 µm^2^, divided into 128 × 128 pixels. Image acquisition comprised 10 scans of the analysed surface, and 10 pulses of Bi^3+^ ions beam were shot at any pixel during each scan. Both spectra and images were acquired with the primary ion dose the below static limit of 1 × 10^13^ ions/cm^2^.

## 3. Results and Discussion

**Figure 1:**
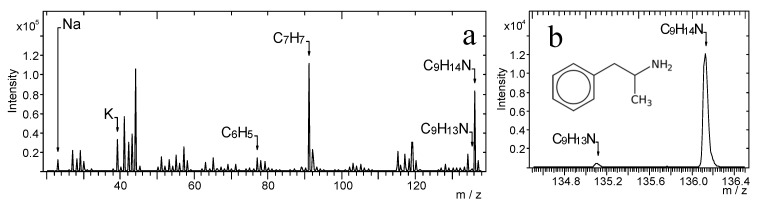
TOF-SIMS positive ion mass spectrum of a tablet of amphetamine: (**a**) *m*/*z* range = 20–138, (**b**) *m*/*z* range = 134.5–136.5.

Figure 1a shows the TOF-SIMS spectrum of amphetamine with characteristic peaks at *m*/*z* 135.10 and 136.12 corresponding to the molecular (C_9_H_13_N^+^) and protonated ion of amphetamine (C_9_H_14_N^+^), respectively. There are also two peaks of characteristic ions, which are the products of fragmentation of the amphetamine molecule: C_6_H_5_^+^ (*m*/*z* = 77.04) and C_7_H_7_^+^ (*m*/*z* = 91.06). These ions were identified as probably the most characteristic fragmentation ions of amphetamine. However, these ions can be also linked with the frequently occurring contamination in samples. The enlarged fragment of the spectrum containing peaks of C_9_H_13_N^+^ and C_9_H_14_N^+^ ions is presented in Figure 1b.

**Figure 2:**
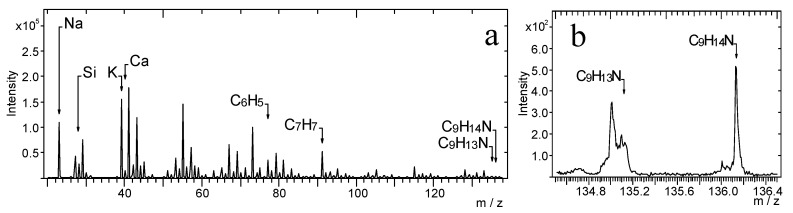
TOF-SIMS positive ion mass spectrum of amphetamine-contaminated fingerprint (on glass surface), (**a**) *m*/*z* range = 20–138, (**b**) *m*/*z* range = 134.5–136.5.

Figure 2 presents the TOF-SIMS spectrum of the amphetamine-contaminated fingerprint that was deposited on the glass. The spectrum in Figure 2b (*m*/*z*) shows peaks characteristic of amphetamine (C_9_H_14_N, C_9_H_14_N^+^), whose position is consistent with the location of peaks recorded for the amphetamine sample pressed into a tablet (Figure 1) and treated in our research as a reference sample. Figure 2b shows that the peak of the molecular ion of amphetamine overlaps with the peak of the ion with *m*/*z* = 135.05, which is generated from the fingerprint support. Therefore, we consider the C_9_H_14_N^+^ ion as the most characteristic peak of the amphetamine molecule.

**Figure 3:**
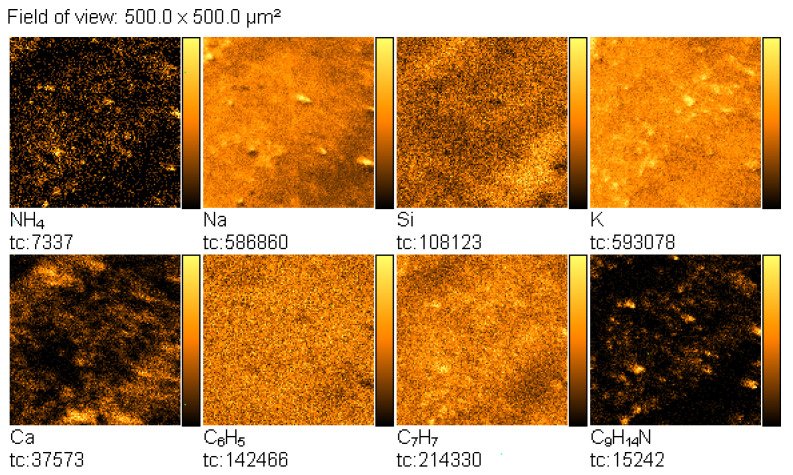
TOF-SIMS positive ion images (500 × 500 µm^2^) of fingerprint contaminated by amphetamine (deposited on glass), showing tc—total counts.

Then, the TOF-SIMS ion imaging was used to trace the microparticles of amphetamine in the fingerprint deposited on the glass support (Figure 3).

NH_4_^+^, C_6_H_5_^+^, C_7_H_7_^+^, and C_9_H_14_N^+^ ions were selected as indicative of amphetamine, and NH_4_^+^, Na^+^, and K^+^ ions were chosen as characteristic sweat. The emission intensity of these ions was not uniform, showing spatial distribution, which reflects the morphology of the analysed fingerprint. In this mode of TOF-SIMS operation, the higher the intensity of secondary ions emission from the analysed area, the brighter this area in the corresponding image.

According to the standard procedure in the forensic science, fingerprints are transferred from the original base with lift tapes. Therefore, we find it necessary to verify whether the TOF-SIMS technique is able to detect amphetamine on the fingerprints imprinted on the lift tape. In the preliminary study, we analysed fingerprints deposited directly on the surface of the lift tape. Three different lift tapes were used for analysis.

**Figure 4:**
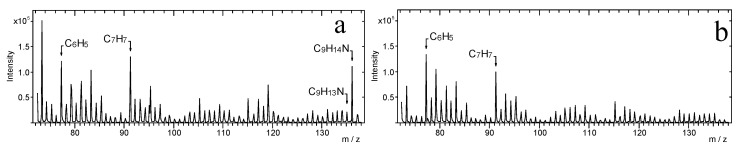
TOF-SIMS positive ion mass spectra (*m*/*z* range 72–138) of: (**a**) Scotch tape with contaminated by amphetamine fingerprint and (**b**) clean Scotch tape.

Figure 4a presents the TOF-SIMS spectrum of the fragment of the fingerprint left on Scotch tape. In this spectrum, a C_9_H_14_N^+^ ion peak is clearly visible. The other two peaks attributed to the fragmentation ions of the amphetamine molecule, at *m*/*z* = 77.04 (C_6_H_5_^+^) and *m*/*z* = 91.06 (C_7_H_7_^+^), could not be used as an amphetamine marker because they were also found in the spectrum of the clean tape (Figure 4b).

**Figure 5:**
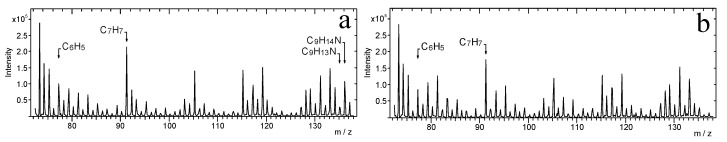
TOF-SIMS positive ion mass spectra (*m*/*z* range 72–138) of: (**a**) Filmolux film with amphetamine contaminated fingerprint and (**b**) clean Filmolux film.

**Figure 6:**
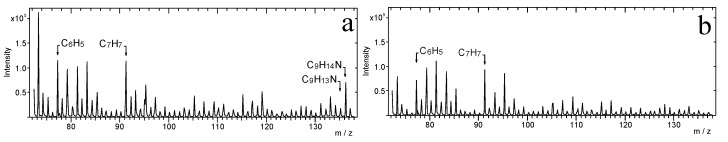
TOF-SIMS positive ion mass spectra (*m*/*z* range 72–138) of: (**a**) Remco tape with contaminated by amphetamine fingerprint and (**b**) clean Remco tape.

A similar analysis was carried out for Filmolux film and Remco tape, and the corresponding spectra are presented in Figure 5 and Figure 6, respectively. The presence of the peaks of C_9_H_13_N^+^ and C_9_H_14_N^+^ ions is observed in all spectra of contaminated tapes, indicating the existence of amphetamine in the fingerprints.

**Figure 7:**
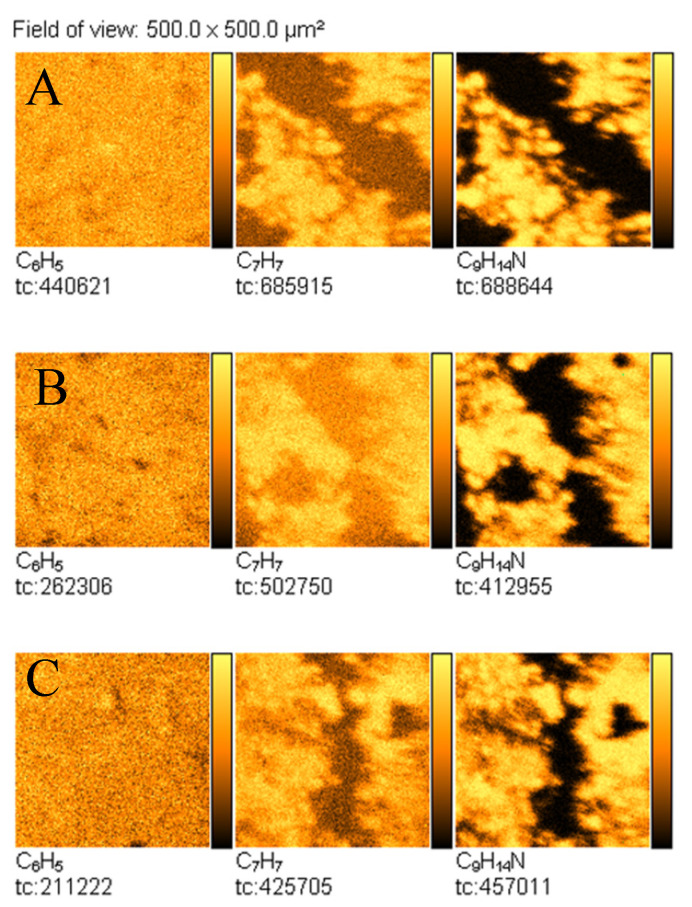
TOF-SIMS positive ion images (500 × 500 µm^2^) of fingerprint contaminated by amphetamine taken from: (**A**) Scotch tape, (**B**) Filmolux film, and (**C**) Remco tape surfaces.

**Figure 8:**
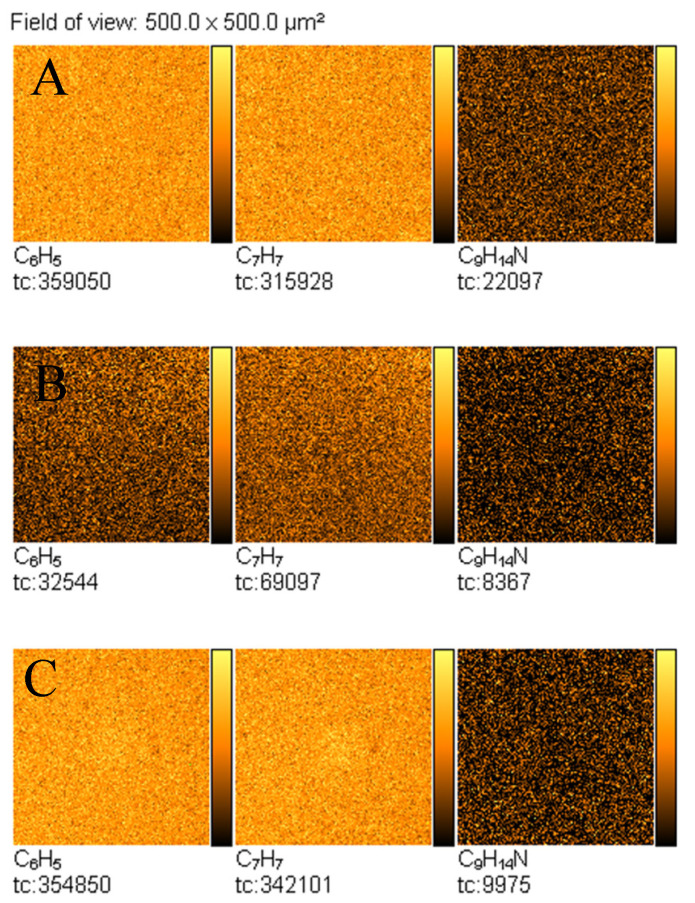
TOF-SIMS positive ion images (500 × 500 µm^2^) of adhesive materials of new, unused tape, taken from: (**A**) Scotch tape, (**B**) Filmolux film, and (**C**) Remco tape surfaces.

Furthermore, TOF-SIMS images of the surface of the analysed samples were recorded. Figure 7 shows TOF-SIMS images of the emission of three characteristic secondary ions—C_6_H_5_^+^, C_7_H_7_^+^, and C_9_H_14_N^+^—from the selected areas of fingerprints contaminated by amphetamine. It is noteworthy that surface distribution of C_6_H_5_^+^, C_7_H_7_^+^, and C_9_H_14_N^+^ ions emission from the tapes follows the pattern of the fingerprint. It should be noted that there is no emission of the C_9_H_14_N^+^ ion from the clean tapes, whereas peaks of the C_6_H_5_^+^ and C_7_H_7_^+^ ions are also observed from the clean tapes, as shown in Figure 8.

Due to the large number of analysed objects, one product was selected from the three tested adhesive materials (Scotch tape, Filmolux film, Remco tape). The chosen Remco tape was characterised by the best quality of the obtained fingerprints contaminated with amphetamine after being revealed by latent fingerprint powder and further transfer.

The application of various types of powders to reveal fingerprints has long been established as an effective and reliable method for developing latent fingerprints [14]. Fingerprints developed at a crime scene are usually lifted with tapes and then kept in evidence bags for further forensic research. As a result, fingerprints designated for the subsequent chemical analysis usually contain latent fingerprint powder, which may inhibit the detection of the studied substances. Therefore, in this study, we tried to prove that it is possible to detect exogenous substances on fingerprints, drugs, for example, after revealing fingermarks with the latent fingerprint powder.

**Figure 9:**
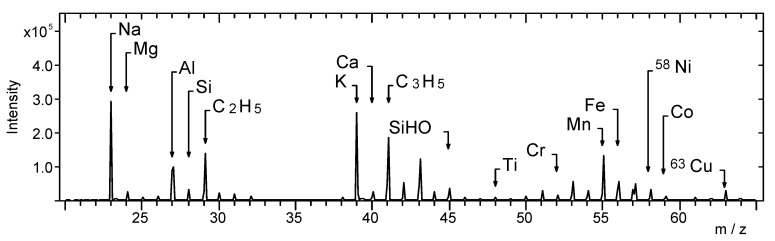
TOF-SIMS positive ion mass spectrum of pastille of black latent fingerprint powder, *m*/*z* range 20–65.

Figure 9 presents the TOF-SIMS spectrum of the black latent fingerprint powder used in this study. It can be seen that Na, Mg, Al, Si, K, Ca, Ti, Cr, Mn, Fe, Ni, Co, and Cu are the main elemental components of this powder. The C_2_H_5_^+^ and C_3_H_5_^+^ ions in the spectrum can be attributed to the possible organic compounds contained in the latent fingerprint powder.

**Figure 10:**
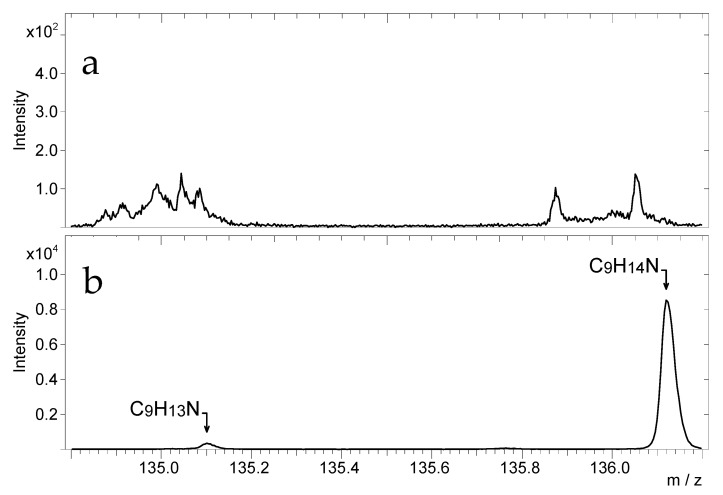
TOF-SIMS positive ion mass spectra of (**a**) the pastille of black latent fingerprint powder and (**b**) pastille of amphetamine; *m*/*z* range 134.8–136.2.

Figure 10 presents the TOF-SIMS spectra of the latent fingerprint powder (Figure 10a) and a tablet of amphetamine (Figure 10b) in the *m*/*z* range from 134.8 to 136.2.

This comparison shows that the spectrum of the latent fingerprint powder does not contain peaks overlapping with those of amphetamine (C_9_H_13_N^+^) and protonated amphetamine (C_9_H_14_N^+^).

**Figure 11:**
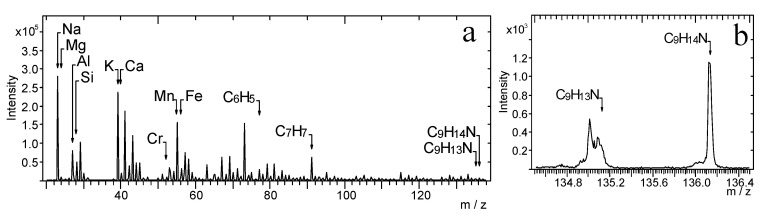
TOF-SIMS positive ion mass spectrum of fingerprint contaminated by amphetamine revealed with black latent fingerprint powder: (**a**) *m*/*z* range 20–138, (**b**) *m*/*z* range 134.5–136.5.

Figure 11 presents the TOF-SIMS spectrum of a fingerprint contaminated by amphetamine, which was visualised with the black latent fingerprint powder on a non-porous surface. It is known that the black powder may contain ferric oxide and rosin, manganese dioxide and rosin, titanium dioxide, quartz, kaolin, inorganic salts, lamp black, and carbon soot.

Both the ions characteristic of the powder and amphetamine are visible in the spectrum. The major peak of the amphetamine ion in Figure 11a has low intensity; therefore, it is presented separately in Figure 11b.

**Figure 12:**
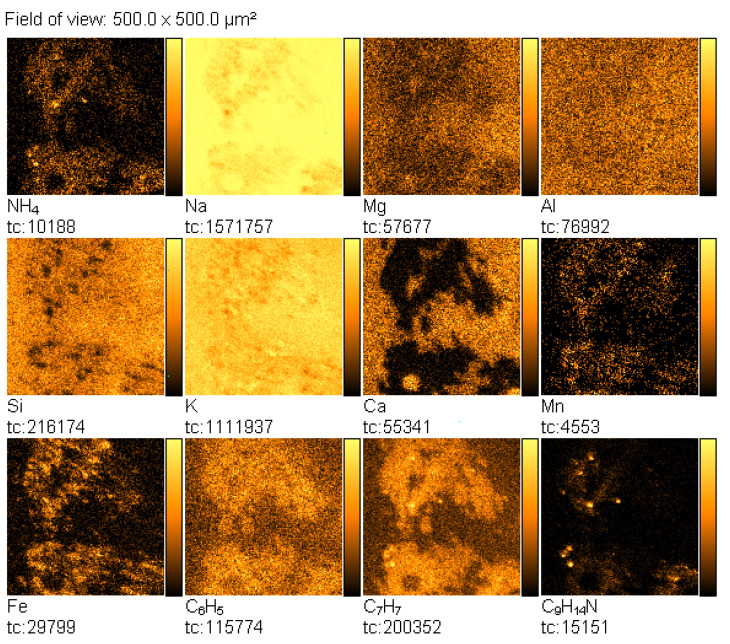
TOF-SIMS positive ion images (500 × 500 µm^2^) of fingerprint contaminated by amphetamine revealed with black latent fingerprint powder (deposited on glass surface).

TOF-SIMS images of the fingerprints contaminated by amphetamine revealed with the black latent fingerprint powder are presented in Figure 12. From these images, it can be seen that spatial distribution of the emission of the NH_4_^+^, C_6_H_5_^+^, C_7_H_7_^+^, and C_9_H_14_N^+^ ions coincides with those characteristic of the fingerprint powder components (Mn^+^, Fe^+^). Na^+^, Ca^+^, and Mg^+^ ions that may originate from another source, e.g., a glass base, are overlapped by those originating from amphetamine and the fingerprint powder. On the other hand, the same spatial distribution of NH_4_^+^, C_6_H_5_^+^, C_7_H_7_^+^, and C_9_H_14_N^+^ ions strongly proves that these ions really originate from amphetamine. Moreover, it can be concluded from Figure 12 that amphetamine and fingerprint powder originate from the same adhesive spot on the finger.

**Figure 13:**
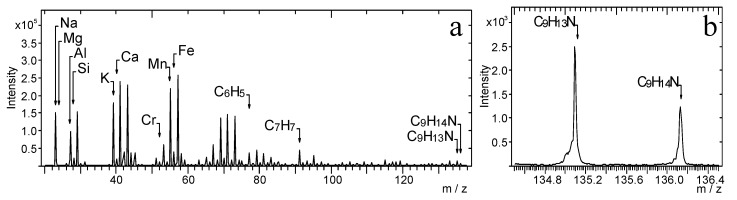
TOF-SIMS positive ion mass spectrum of fingerprint contaminated by amphetamine revealed with black latent fingerprint powder and transferred from glass surface using Remco tape, (**a**) *m*/*z* range = 20–138, (**b**) *m*/*z* range = 134.5–136.5.

Moreover, TOF-SIMS was also successfully applied to detect amphetamine residues on the fingerprints visualised by the powder and transferred on the tape, as it is evidenced by the spectrum in Figure 13. At this step, only one type of the tested lift tapes was used: Remco tape.

**Figure 14:**
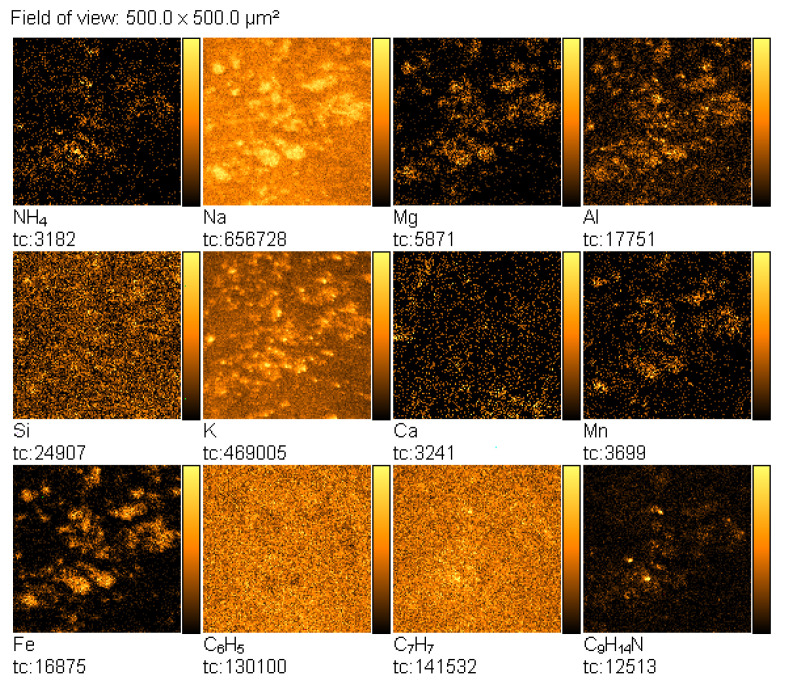
TOF-SIMS positive ion images (500 × 500 µm^2^) of fingerprint contaminated by amphetamine revealed with black latent fingerprint powder and transferred from glass surface using Remco tape.

Figure 14 presents TOF-SIMS images of fingerprints containing a trace amount of amphetamine revealed with the black latent fingerprint powder and transferred onto the tape. Similarly, as in the case of the non-transferred fingerprints, emission of the Mn^+^ and Fe^+^ ions correlates spatially with the emission of the NH_4_^+^, C_6_H_5_^+^, C_7_H_7_^+^, and C_9_H_14_N^+^ ions, which are characteristic of amphetamine.

## 4. Conclusions

The results demonstrated that TOF-SIMS can be helpful in obtaining fragments of the morphological characteristics of the latent fingerprints and the simultaneous detection of exogenous substances, which can be present on the suspect’s fingers. TOF-SIMS imaging of the studied fingerprints made it possible to gather information about the distribution of chemicals of detected components on fingerprints. This, combined with the spectra analysis, made it possible to correlate the presence of specific ions with their possible source.

It should also be emphasised that the images obtained by the TOF-SIMS technique enable the observation of very small areas of the analysed fingerprint as well as the identification of micro-objects left on the fingerprint (residues of the contaminant). Therefore, in the application of TOF-SIMS in forensic investigation, the sample of a fingerprint left on an object must be prepared in the form and size appropriate for the TOF-SIMS technique.

Moreover, it was shown that revealing fingerprints with the black fingerprint powder is not disadvantageous for the TOF-SIMS application in the fingerprint analysis. Although the measurement was slightly less effective, a good MS image could be obtained with the latent material.

The TOF-SIMS technique appears to be a very effective and useful tool in forensic investigations, which enables the identification of drug residues left on fingerprints imprinted on selected substrates. Even though the powder presence clearly limits the effectiveness of the analysis, the potential of the TOF-SIMS technique in the identification and detection of amphetamine has been confirmed, which seems to be valuable information for the forensic analysis.

## Data Availability

Not applicable.
